# Post-Transcriptional Regulation of PARP7 Protein Stability Is Controlled by Androgen Signaling

**DOI:** 10.3390/cells10020363

**Published:** 2021-02-09

**Authors:** Teddy Kamata, Chun-Song Yang, Tiffany A. Melhuish, Henry F. Frierson Jr., David Wotton, Bryce M. Paschal

**Affiliations:** 1 Center for Cell Signaling, University of Virginia School of Medicine, Charlottesville, VA 22908, USA; ttk3pf@virginia.edu (T.K.); cy5y@virginia.edu (C.-S.Y.); tam6c@virginia.edu (T.A.M.); dw2p@virginia.edu (D.W.); 2 Department of Biochemistry and Molecular Genetics, University of Virginia School of Medicine, Charlottesville, VA 22908, USA; 3 Department of Pathology, University of Virginia School of Medicine, Charlottesville, VA 22908, USA; hff@virginia.edu

**Keywords:** ADP-ribosylation, mono-ADP-ribosyltransferase, PARP7, ARTD14, TIPARP, androgen receptor, prostate cancer, protein stability, protein degradation

## Abstract

Poly-ADP-ribose polymerases (PARPs) are enzymes that catalyze ADP-ribosylation and play critical roles in normal and disease settings. The PARP family member, PARP7, is a mono-ADP-ribosyltransferase that has been suggested to play a tumor suppressive role in breast, ovarian, and colorectal cancer. Here, we have investigated how androgen signaling regulates PARP7 homeostasis in prostate cancer cells, where PARP7 is a direct target gene of AR. We found that the PARP7 protein is extremely short-lived, with a half-life of 4.5 min. We show that in addition to its transcriptional regulation by AR, PARP7 is subject to androgen-dependent post-transcriptional regulation that increases its half-life to 25.6 min. This contrasts with PARP1, PARP2, PARP9, and PARP14, which do not display rapid turnover and are not regulated by androgen signaling. Androgen- and AR-dependent stabilization of PARP7 leads to accumulation in the nucleus, which we suggest is a major site of action. Mutations in the catalytic domain, the Cys3His1 zinc finger, and WWE (tryptophan–tryptophan–glutamate) domains in PARP7 each reduce the degradation rate of PARP7, suggesting the overall structure of the protein is tuned for its rapid turnover. Our finding that PARP7 is regulated by AR signaling both transcriptionally and post-transcriptionally in prostate cancer cells suggests the dosage of PARP7 protein is subject to tight regulation.

## 1. Introduction

Poly-ADP-ribose polymerases (PARPs, also known as ADP-ribosyltransferase diphtheria toxin-like (ARTD)) are enzymes that catalyze the covalent attachment of ADP-ribose onto substrates (referred to as ADP-ribosylation) [1,2,3]. This type of post-translational modification occurs in two general forms: mono-ADP-ribosylation where a site is modified by a single ADP-ribose, or poly-ADP-ribosylation where a mono-ADP-ribosylated site is elongated into a polymer of ADP-ribose units through successive rounds of ADP-ribosylation [4,5]. The 17 PARPs encoded by the human genome contain homologous catalytic domains [6]. The majority of these enzymes have been shown biochemically to act as mono-ADP-ribosyltransferases [1,7]. Compared to the founding member PARP1 and poly-ADP-ribosylation, relatively less is known about the functional roles of the mono-ADP-ribosyltransferases and mono-ADP-ribosylation [1,8]. PARPs and ADP-ribosylation play diverse roles in human biology including transcription regulation, DNA damage repair, metabolism, mitosis, cell signaling, and the immune response [2,9,10,11,12]. The development of PARP inhibitors that target PARP1 in the context of DNA damage signaling and cancer has impacted clinical practice [13], and suggests that additional opportunities remain for developing new therapeutics. Inhibitor development can also shed light on the roles of other PARPs and mono-ADP-ribosylation mechanisms in normal physiology and disease processes.

PARP7 (also known as 2,3,7,8-tetrachlorodibenzo-*p*-dioxin-inducible poly-ADP-ribose polymerase [TIPARP] or ARTD14), is a member of the PARP enzyme family and is classified as a mono-ADP-ribosyltransferase [1,7]. PARP7 has been studied mostly in the context of aryl hydrocarbon receptor (AHR) signaling in liver during dioxin toxin exposure [14]. In this setting, PARP7 is induced by an AHR ligand and acts as a negative regulator of AHR by mono-ADP-ribosylating the receptor and suppressing AHR-dependent gene transcription [15,16]. In liver cells, PARP7 can also mono-ADP-ribosylate the liver X receptors (LXR), but in this signaling pathway, it positively regulates LXR-dependent gene transcription [17]. Aside from transcription regulation, PARP7 also has immune-modulatory functions, and both positive and negative roles have been described in the context of viral infections. PARP7 can be induced by viral infection and suppress viral replication by downregulating protein translation globally [18,19] or targeting viral RNA for degradation [20]. Finally, induction of PARP7 by AHR during viral infection leads to downregulation of type I IFN response [21].

In addition to regulation of transcription and the immune response, there is growing evidence that PARP7 may play a key role in cancer. *PARP7* mRNA and protein expression were decreased in breast cancer cells [22], and *PARP7* knockdown in MCF7 breast cancer cells prevented the establishment of tumors in xenograft models, indicating a tumor suppressive role for PARP7 [23]. Stratification of breast cancer patients based on *PARP7* mRNA expression level showed a significant survival benefit for patients expressing higher levels of *PARP7*, further underscoring a potential tumor suppressive role for PARP7 [23]. This trend for PARP7 also appears to hold true in ovarian cancer. A single nucleotide polymorphism was identified in the *PARP7* gene that increases the risk of ovarian cancer, and models for ovarian cancer progression showed that *PARP7* gene expression decreased with neoplastic development [24]. In support of these observations, amplification of the *PARP7* gene and presumed higher gene expression, was associated with significant survival benefits in ovarian cancer patients [25]. Lastly, overexpression of *PARP7* in a xenograft model for HCT116 colorectal cancer slowed tumor growth, while knockdown of *PARP7* showed the opposite effect [23]. Thus, in three cancer types, the data point toward PARP7 exerting tumor suppressive effects. Aside from PARP7 being established as a direct androgen receptor (AR) target gene [26], very little is known about PARP7 in prostate cancer (PCa) where AR signaling plays a major role, and we set out to explore the interplay between AR signaling and PARP7 in this context. Here, we show that in PCa cells PARP7 is rapidly degraded by the proteasome, and that AR signaling stabilizes PARP7 and leads to protein accumulation in the nucleus.

## 2. Materials and Methods

### 2.1. Plasmid DNA

N-terminally 3xHA-tagged PARP7 wild-type (WT) CDS was cloned into a custom lentiviral Tet-On inducible vector carrying a puromycin selectable marker (TetON-HA-PARP7) or pWPI lentiviral vector (Addgene, Watertown, MA, USA, plasmid #12254) carrying an EGFP marker (pWPI/HA-PARP7). N-terminally Avi-tagged PARP7 WT CDS was cloned into a custom lentiviral vector carrying a hygromycin selectable marker (pL-Hyg-Avi-PARP7). N-terminally HA-tagged WT or V582F mutant AR CDS was cloned into a custom lentiviral vector carrying a puromycin selectable marker (pL-Puro-HA-AR). Site-directed mutagenesis was conducted on TetON-HA-PARP7 lentiviral vector to generate C243A, C251A, H532A, and Y564A PARP7 mutants. A deletion of the WWE (tryptophan-tryptophan-glutamate) domain of PARP7 (amino acids: 332 to 401, ∆WWE) was achieved by overlap extension PCR and cloned into the TetON lentiviral vector.

### 2.2. Cell Lines

PC3 (prostate cancer) cells (RRID:CVCL_0035) and PC3M (metastasis-derived variant of PC3, RRID:CVCL_9555) were kindly provided by Dr. Michael Weber (University of Virginia, Charlottesville, VA, USA). PC3 cells stably expressing N-terminally Flag-tagged AR (PC3-Flag-AR) were generated previously [27]. PC3 cells stably expressing Flag-tagged AR and HA-tagged PARP7 (PC3-Flag-AR/HA-PARP7) was generated from PC3-Flag-AR cells via lentiviral transduction with the pWPI/HA-PARP7 vector and cell sorting using an EGFP marker. PC3 cells stably expressing Flag-tagged AR and doxycycline-inducible HA-tagged PARP7 WT or mutant (PC3-Flag-AR/TetON-HA-PARP7) were generated from PC3-Flag-AR cells via lentiviral transduction with the TetON-HA-PARP7 vector (WT, C243A, C251A, H532A, Y564A, or ∆WWE) and maintained under selection with 1 µg/mL puromycin. PC3M cells stably expressing HA-tagged AR (WT or V582F) and Avi-tagged-PARP7 was generated via lentiviral transduction and maintained under selection with 1 µg/mL puromycin and 0.2 mg/mL hygromycin. PC3 and its cell line derivatives were all grown in RPMI 1640 medium supplemented with 5% fetal bovine serum (Cytiva, Marlborough, MA, USA; SH30396.03HI) and 100 U/mL penicillin/streptomycin (Thermo Fisher Scientific, Waltham, MA, USA). VCaP (prostate cancer) cells (RRID:CVCL_2235) were maintained in DMEM/F12 (1:1) medium supplemented with 5% fetal bovine serum (Cytiva) and 100 U/mL penicillin/streptomycin (Thermo Fisher Scientific). All cells were cultured at 37 °C with 5% CO_2_.

### 2.3. Chemical Reagents

MG132, DRB, cycloheximide, triptolide, and enzalutamide (ENZ) were purchased from Cayman Chemical Company (Ann Arbor, MI, USA). R1881 (methyltrienolone) was purchased from PerkinElmer, Inc. (Waltham, MA, USA). Bicalutamide was purchased from Thermo Fisher Scientific. Dihydrotestosterone (DHT), androstenedione (ASD), dehydroepiandrosterone (DHEA), flutamide, hydroxyflutamide (HO-Flut), and cyproterone acetate (CPA) were purchased from Sigma-Aldrich (St. Louis, MO, USA).

### 2.4. Antibodies

Anti-AR antibody (custom rabbit antibody raised against AR residues 1 to 21: MEVQLGLGRVYPRPPSKTYRGC), anti-PARP7 antibody (custom rabbit antibody raised against PARP7 residues 119 to 132: DQIPEAHPSTEAPE), anti-PARP9 antibody (custom rabbit antibody raised against PARP9 catalytic domain), and anti-FKBP51 antibody (custom rabbit antibody raised against full-length protein) were generated by Cocalico Biologicals, Inc. (Stevens, PA, USA). Anti-PARP14 mouse monoclonal antibody (sc-377150) was purchased from Santa Cruz Biotechnology, Inc. (Dallas, TX, USA). Anti-PARP1 rabbit monoclonal antibody (ab32138), anti-PARP2 rabbit monoclonal antibody (ab176330), and anti-Histone H3 rabbit polyclonal antibody (ab1791) were purchased from Abcam (Cambridge, United Kingdom). Anti-HA (16B12) mouse monoclonal antibody was purchased from Covance (Princeton, NJ, USA). Anti-tyrosine tubulin (clone TUB-1A2) mouse monoclonal antibody was purchased from Sigma-Aldrich. The following secondary antibodies for immunoblotting were purchased: IRDye^®^ 800-conjugated goat anti-mouse IgG (Rockland Immunochemicals, Inc., Limerick, PA, USA, 610-132-121) and AlexaFluor^®^ 680-conjugated donkey anti-rabbit IgG (Thermo Fisher Scientific, A10043). For immunofluorescence microscopy, Cy3-labeled donkey anti-mouse (715-165-151) and Cy5-labeled donkey anti-rabbit (711-175-152) secondary antibodies were purchased from Jackson ImmunoResearch Laboratories, Inc. (West Grove, PA, USA).

### 2.5. Immunoblotting

Whole cell extracts were prepared by lysing cells in 1× sample buffer and were resolved by SDS-PAGE. After transfer, nitrocellulose membranes with immobilized proteins were blocked for at least 1 h with blocking solution (5% nonfat dry milk (*w*/*v*)/1× PBS with 0.15% Tween 20 (*v*/*v*)), followed by primary, and then, secondary antibody incubation. Membranes were imaged on Odyssey^®^ CLx imaging system (LI-COR Biosciences, Lincoln, NE, USA), and quantification of protein bands were done in the associated instrument software Image Studio Lite version 5.2.5 (LI-COR Biosciences).

### 2.6. Protein Half-Life Determination

Cells were plated in 35 mm tissue culture dishes at least 48 h before conducting a cycloheximide time course treatment. Cycloheximide was added to the culture medium at a final concentration of 100 µg/mL to stop protein synthesis. After addition of cycloheximide, cells were harvested in 1× sample buffer at various timepoints and analyzed by SDS-PAGE and immunoblotting. Protein bands were quantified on the Odyssey^®^ CLx imaging system and normalized by tubulin. Normalized protein expression levels were plotted on a natural log-linear plot, and the decay constant (k) was derived from the linear fit. Protein half-life was calculated using the formula t_1/2_ = ln(2)/k. Time course experiments were repeated three times to determine each protein half-life.

### 2.7. RT-qPCR

Total RNA was isolated from cells using RNeasy kit (QIAGEN, Hilden, Germany), according to manufacturer’s protocol. cDNA was prepared from 1 µg of RNA using iScript cDNA synthesis kit (Bio-Rad Laboratories, Hercules, CA, USA). qPCR was conducted using SensiMix™ SYBR^®^ and Fluorescein kit (Bioline, London, United Kingdom; QT615-05). The following primer pairs were used: HA-PARP7 (5′- CTAGCGCCACCATGTACCC-3′ and 5′- GGTTCGGTGGTTTCCATTTCG-3′), and *GUS* (5′-CCGACTTCTCTGACAACCGACG-3′ and 5′-AGCCGACAAAATGCCGCAGACG-3′). Gene expression was normalized against the housekeeping gene *GUS*, and the mean and standard deviation were calculated from three biological replicates.

### 2.8. Immunofluorescence Microscopy

Cells were seeded onto glass coverslips at least 48 h prior to fixation. After drug treatment, cells were washed three times in 1× PBS and fixed in 3.75% formaldehyde/1× PBS for 15 min. Coverslips were washed three times in 1× PBS and incubated in 0.2% Triton X-100/1× PBS for five minutes to permeabilize cells. Afterwards, coverslips were washed three times in 1× PBS and blocked for one hour at room temperature in blocking buffer (2% BSA/1× PBS). Coverslips were then incubated overnight at 4 °C in primary antibodies diluted in blocking buffer, washed three times in 1× PBS, and incubated for one hour at room temperature in secondary antibodies diluted in blocking buffer. Coverslips were washed twice in 1× PBS before incubating 10 s in 1.3 µg/mL DAPI/1× PBS to stain nuclei. A final wash with deionized water was conducted to remove excess buffer salt before mounting on glass slides with VectaShield (Vector Laboratories, Burlingame, CA, USA). Images were acquired on a Nikon Eclipse Ni-U microscope (Nikon Instruments, Inc., Melville, NY, USA) equipped with a DS-Qi1Mc camera at 40× objective and processed using Adobe Photoshop version 21.2.2 (Adobe Inc., San Jose, CA, USA) and Fiji ImageJ version 2.0.0. HA-PARP7 cellular distribution was quantified as a ratio of nuclear (N) to cytoplasmic (C) signal as described previously [28]. Regions of interest were outlined in the nucleus and the cytoplasm, and mean intensities with background signal subtracted were determined before calculating the N/C ratio. At least 100 cells were quantified for each condition.

### 2.9. Mice and Histology

The *loxP* flanked (referred to here as ‘*f*’) *Pten* and the *PbCre4* transgenic are as described [29,30]. *Tiparp* mice (*Tiparp^tm1a(EUCOMM)Wtsi^*) were generated from cryopreserved sperm (from the Canadian Mutant Mouse Repository, Hospital for Sick Children, Toronto) by IVF at the University of Virginia GEMM Core. The conditional *Tiparp* allele was generated from *Tiparp^tm1a(EUCOMM)Wtsi^* by crossing to a Flpo mouse (Jax 12930; [31]) to remove the *SA-lacZ* and *Neo* cassette. Mice were maintained on a mixed strain background (C57BL/6 × FVB), genotyped and analyzed as previously described [32,33]. Prostates were fixed in zinc-formalin, paraffin embedded, and sectioned at 5 microns, and were stained with hematoxylin and eosin (H&E). Images were captured with 10 or 20× objectives, using a Nikon Eclipse Ni-U microscope with a DS-Ri1 camera and NIS Elements software version 4.13 (Nikon Instruments, Inc.), and adjusted in Adobe Photoshop.

### 2.10. Statistical Analysis

Statistical analysis was conducted in GraphPad Prism version 9.0.1 software (GraphPad Software, San Diego, CA, USA). To determine statistical significance, unpaired t-test or one-way ANOVA with Tukey’s multiple comparison test was conducted as appropriate.

### 2.11. Ethics Statement

All animal procedures were approved by the Animal Care and Use Committee of the University of Virginia, which is fully accredited by the AAALAC, and were carried out under protocol #3829 (approved on the 14 May 2020).

## 3. Results

### 3.1. PARP7 Is Regulated by a Post-Transcriptional Mechanism that Requires AR

**Figure 1:**
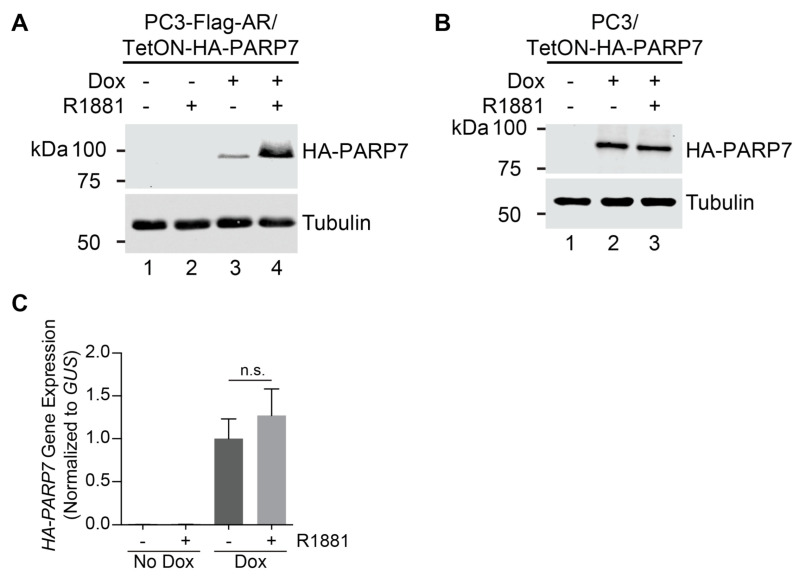
Androgen receptor (AR) signaling can increase PARP7 protein level independent of *PARP7* transcript level. (**A**) PC3 cells stably expressing EF1α-driven Flag-tagged AR and doxycycline-inducible HA-tagged PARP7 (PC3-Flag-AR/TetON-HA-PARP7) were treated with doxycycline (2 µg/mL Dox, 24 h) followed by androgen (2 nM R1881, 6 h) and analyzed by immunoblotting. (**B**) PC3 cells stably expressing doxycycline-inducible HA-tagged PARP7 (PC3/TetON-HA-PARP7) were treated as in (**A**) and analyzed by immunoblotting. (**C**) PC3-Flag-AR/TetON-HA-PARP7 cells were treated as in (**A**), and *HA-PARP7* mRNA transcript level was determined by RT-qPCR. Gene expression was normalized to the housekeeping gene *GUS*. Plot shows mean ± SD for three biological replicates, and vehicle- and R1881-treated samples from the Dox group were compared by unpaired t-test (n.s.—not significant).

AR ChIP-on-chip analysis combined with an androgen signaling microarray indicate that *PARP7* is a direct target gene for AR in normal prostate epithelial cells [26]. Complementing these findings are our RNA-seq data which show that *PARP7* is induced by the synthetic androgen R1881 in multiple PCa lines, including a PC3 cell line engineered to express wild-type (WT) AR [27]. PC3 cells, which were derived originally from a bone metastasis in a PCa patient, are one of the most aggressive models of PCa [34,35]. We engineered PC3-Flag-AR cells for AR-independent HA-PARP7 induction using the TetON system. By immunoblotting, HA-PARP7 is detected only after addition of Dox (Figure 1A, lane 3). Unexpectedly, co-treatment of the cells with Dox and R1881 led to a substantial increase in the level of HA-PARP7 (Figure 1A, lane 4). The R1881 effect on HA-PARP7 protein level was not observed in PC3 cells that lack AR (Figure 1B). Moreover, in the AR-positive cells, R1881 treatment did not significantly affect *HA-PARP7* message levels induced by Dox treatment (Figure 1C). These data provide clear evidence that PARP7 protein levels can be modulated post-transcriptionally by an androgen-dependent mechanism that requires AR.

### 3.2. PARP7 Is a Short-Lived Protein that Is Stabilized by AR Signaling

**Figure 2:**
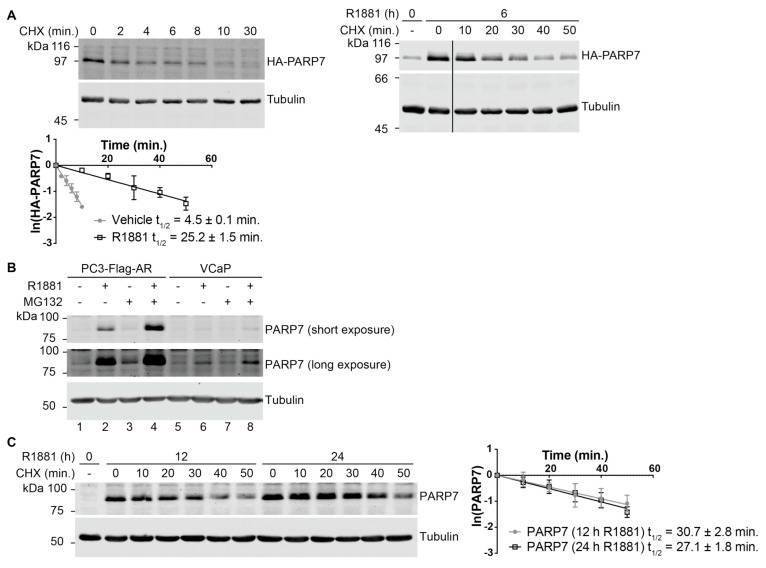
PARP7 undergoes very rapid protein turnover and is stabilized by AR signaling. (**A**) PC3-Flag-AR/HA-PARP7 cells were treated with 100 μg/mL cycloheximide (CHX) for indicated times with or without androgen (2 nM R1881) treatment and analyzed by immunoblotting. HA-PARP7 protein levels were normalized by tubulin and plotted against time on a natural log-linear plot (n = 3, mean ± SD). (**B**) PC3-Flag-AR and VCaP cells were treated for 16 h with androgen (2 nM R1881) followed by 1 h of MG132 (10 µM) and analyzed by immunoblotting. (**C**) PC3-Flag-AR cells were treated for 12 or 24 h with androgen (2 nM R1881) and analyzed as in (**A**) to determine the protein half-life for PARP7. Plots show mean ± SD (n = 3) and protein half-lives.

The positive effect of R1881 on HA-PARP7 protein levels led us to hypothesize that AR signaling modulates the turnover mechanism of PARP7. To formally test this hypothesis, we measured the half-life of HA-PARP7 using the protein synthesis inhibitor cycloheximide (CHX) in untreated and R1881-treated cells. Under untreated (basal) conditions, we found that HA-PARP7 has a very short half-life (4.5 ± 0.1 min), and that R1881 treatment increases the half-life approximately 5.6-fold (25.2 ± 1.5 min; Figure 2A). PARP7 degradation is proteasome-dependent based on the fact that MG132 treatment increased endogenous PARP7 levels after its expression was induced by androgen (Figure 2B). Furthermore, we examined PARP7 in another PCa cell line, VCaP, and detected PARP7 expression at lower levels than in PC3-Flag-AR cells (Figure 2B, compare lane 2 and 6), which is consistent with the lower RNA counts observed for *PARP7* in VCaP cells based on our RNA-seq datasets [27]. As in PC3-Flag-AR cells, MG132 treatment in VCaP cells increased the amount of PARP7 suggesting that PARP7 rapid turnover by the proteasome is a general characteristic of PCa cells (Figure 2B, compare lane 6 and 8). To determine the protein half-life of endogenous PARP7, we treated PC3-Flag-AR cells with R1881 for 12 and 24 h, chased with CHX, and examined PARP7 protein levels using an affinity-purified antibody. The half-life of endogenous PARP7 was 30.7 ± 2.8 and 27.1 ± 1.8 min in cells treated with androgen for 12 and 24 h, respectively (Figure 2C). Thus, our data supports the model that PARP7 is a rapidly degraded protein that can be stabilized by androgen signaling through the AR, resulting in PARP7 protein accumulation.

### 3.3. Multiple Domains Contribute to the Rapid Turnover of PARP7

**Figure 3:**
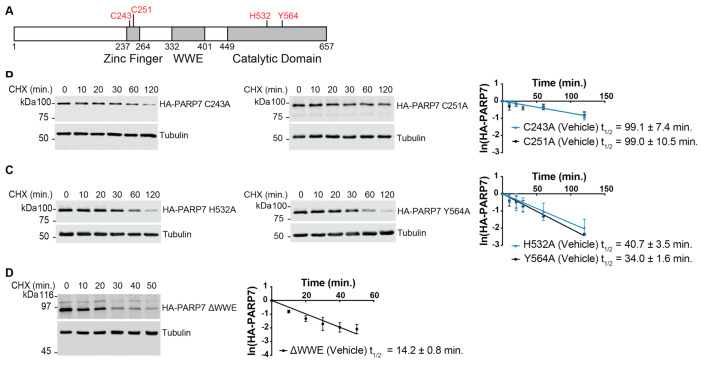
Zinc finger, tryptophan–tryptophan–glutamate (WWE), and catalytic domain contribute to the very rapid turnover properties of PARP7. (**A**) PARP7 protein structure. Point mutants used in the analysis are indicated in red. (**B**) PARP7 zinc finger mutants (C243A or C251A), (**C**) catalytic mutants (H532A or Y564A), or (**D**) WWE deletion mutant (∆WWE) was induced in PC3-Flag-AR cells by doxycycline treatment (2 μg/mL, 24 h). Cycloheximide (CHX) time course experiments were conducted and analyzed as described in Figure 2A. Plots show mean ± SD (n = 3) and protein half-lives.

There is rationale to suggest that multiple domains of PARP7 might have roles in its turnover. These include a ubiquitylation site in the zinc finger [36], and targeting functions in the catalytic and WWE domains that direct PARP7 to nuclear bodies where other proteins are degraded [23]. To this end, we generated cell lines expressing PARP7 proteins with single amino acid substitutions in the zinc finger (C243A and C251A) and catalytic domain (H532A and Y564A), as well as a deletion of the WWE domain (amino acids: 332 to 401; Figure 3A). The C243A and C251A substitutions target key cysteine residues that coordinate the structural zinc ion and have been shown to cause loss-of-function for PARP7 in terms of its transcription regulatory role in the AHR and LXR signaling pathways [16,17] The H532A and Y564A substitutions abrogate PARP7 enzyme activity as measured by in vitro auto-ADP-ribosylation assays [16,23].

Point mutations in the zinc finger of PARP7 increased protein half-life significantly in the absence of androgen (C243A, 99.1 ± 7.4 min; C251A, 99.0 ± 10.5 min; Figure 3B, Table 1). Unlike for WT PARP7, R1881 treatment did not increase the half-life of the zinc finger mutants (C243A, 74.9 ± 8.8 min; C251A, 90.3 ± 9.2 min; Supplementary Figure S1, Table 1). Point mutations that inactivate the catalytic domain of PARP7 also increased the protein half-life of PARP7 (H532A, 40.7 ± 3.5 min; Y564A, 34.0 ± 1.6 min), but as was the case with the zinc finger mutants, the catalytic domain mutant protein half-lives were not increased significantly by R1881 (Supplementary Figure S1, Table 1). Finally, deletion of the WWE domain increased the PARP7 half-life (14.2 ± 0.8 min), and in CHX chase experiments, this mutant was not stabilized significantly by R1881 (Supplementary Figure S1, Table 1). From these experiments, we conclude that all three domains contribute to the instability of PARP7.

### 3.4. Rapid Turnover and Androgen-Dependent Stabilization Are Not General Characteristics of PARPs

**Figure 4:**
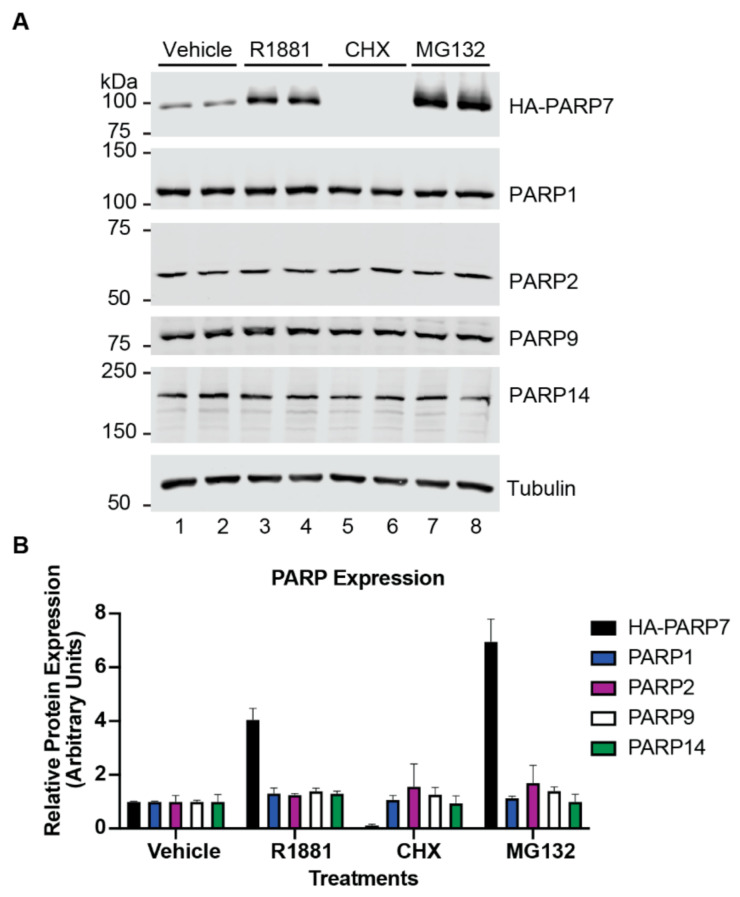
Characterization of androgen regulation and protein turnover of select PARPs in prostate cancer cells. (**A**) PC3-Flag-AR/HA-PARP7 cells were treated with androgen (2 nM R1881, 6 h), cycloheximide (100 µg/mL CHX, 1 h), or MG132 (10 µM, 2 h) and analyzed by immunoblotting. Two independent experiments are shown (odd numbered lanes are from experiment 1, even numbered lanes are from experiment 2). (**B**) Quantification of (**A**). Signal intensities for PARP protein levels were normalized by tubulin and scaled by setting the mean for vehicle treatment to 1. Plot shows mean ± SD from two independent experiments.

We next determined if androgen regulation and protein instability are characteristic of other PARP family members. For these experiments, we used a PC3-Flag-AR cell line with HA-PARP7 expression controlled by the EF1α promoter, which is constitutively active in many cell types. The treatments were carried out in replicate wells, which were subsequently harvested and analyzed by immunoblotting. The basal level of HA-PARP7 expression from the EF1α promoter in this cell line (PC3-Flag-AR/HA-PARP7) permits PARP7 detection by immunoblotting in vehicle treated cells, and as expected, there is a significant increase in HA-PARP7 protein level in response to R1881 (Figure 4A,B; lanes 1–4). CHX treatment (1 h) of the cells resulted in a complete loss of HA-PARP7, consistent with its rapid turnover (Figure 4A,B; lanes 1, 2, 5, 6). MG132 treatment (2 h) led to a large increase in HA-PARP7 protein accumulation, which is indicative of rapid degradation by the proteasome (Figure 4A,B; lanes 1, 2, 7, 8). We then examined the potential effects of these treatments on PARP1, PARP2, PARP9, and PARP14 by immunoblotting the same extracts. None of these PARPs showed substantial response to R1881, CHX, or MG132 under these relatively brief treatments conditions (Figure 4A,B). We conclude that protein instability and post-transcriptional regulation by androgen signaling are not general features of the PARP family.

### 3.5. AR-Dependent Stabilization of PARP7 Drives Its Nuclear Accumulation

**Figure 5:**
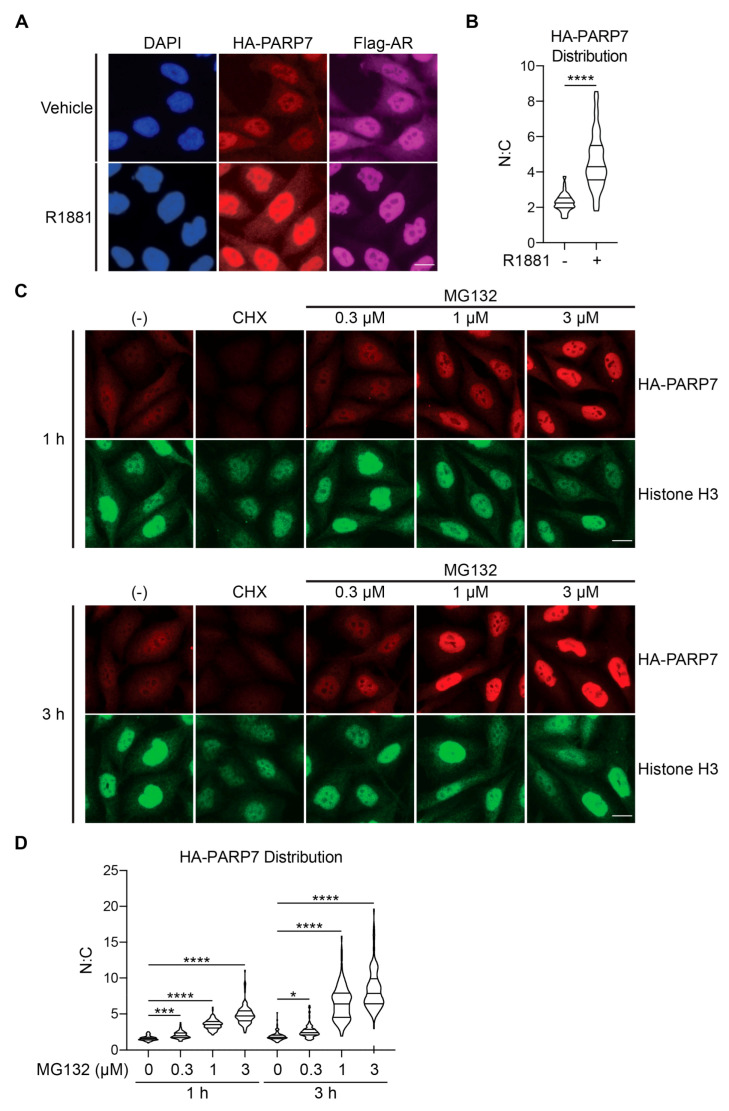
Stabilization of PARP7 by AR signaling leads to nuclear accumulation. (**A**) PC3-Flag-AR/HA-PARP7 cells were treated with androgen (2 nM R1881, 6 h) and processed for immunofluorescence microscopy. Scale bar = 5 µm. (**B**) Quantification of (A). Distribution of HA-PARP7 was quantified as a ratio of nuclear (N) and cytoplasmic (C) signals. At least 100 cells for each condition were analyzed. Violin plot shows median and first and third quartiles. Statistical significance was determined by unpaired t-test (****, *p* < 0.0001). (**C**) PC3-Flag-AR/HA-PARP7 cells were treated with cycloheximide (100 μg/mL CHX) or indicated concentrations of MG132 for 1 or 3 h and processed for immunofluorescence microscopy. Scale bar = 5 µm. (**D**) Quantification of (**C**). Nuclear:cytoplasmic ratio for HA-PARP7 was determined as described in (**B**). At least 100 cells for each condition were analyzed. Violin plot shows median and first and third quartiles. Statistical significance was determined by one-way ANOVA with Tukey’s multiple comparison test (*, *p* < 0.05; ***, *p* < 0.001; ****, *p* < 0.0001).

Previous studies have shown that PARP7 is predominantly nuclear in HuH-7 hepatocarcinoma and embryonic stem cells by virtue of an NLS within the N-terminus [15,16,17,37]. We sought to examine if androgen signaling through AR affects PARP7 subcellular distribution. Prior to R1881 treatment, HA-PARP7 was localized mainly within the nucleus, though a low level of the protein was detected in the cytoplasm by immunofluorescence microscopy (Figure 5A). This generates a mean nuclear:cytoplasmic (N:C) ratio of 2.3. R1881 treatment led to a substantial increase of HA-PARP7 protein in the nucleus, indicated by a statistically significant (*p* < 0.0001) increase in the N:C value for HA-PARP7 (mean: 4.6; Figure 5A,B). The androgen effect on HA-PARP7 accumulation within the nucleus was recapitulated by MG132 treatment in a time and concentration dependent manner (Figure 5C,D). These data indicate that N:C levels of PARP7 are likely regulated by a post-transcriptional mechanism that controls its turnover.

### 3.6. AR-Dependent Transcription Is Necessary for PARP7 Stabilization

**Figure 6:**
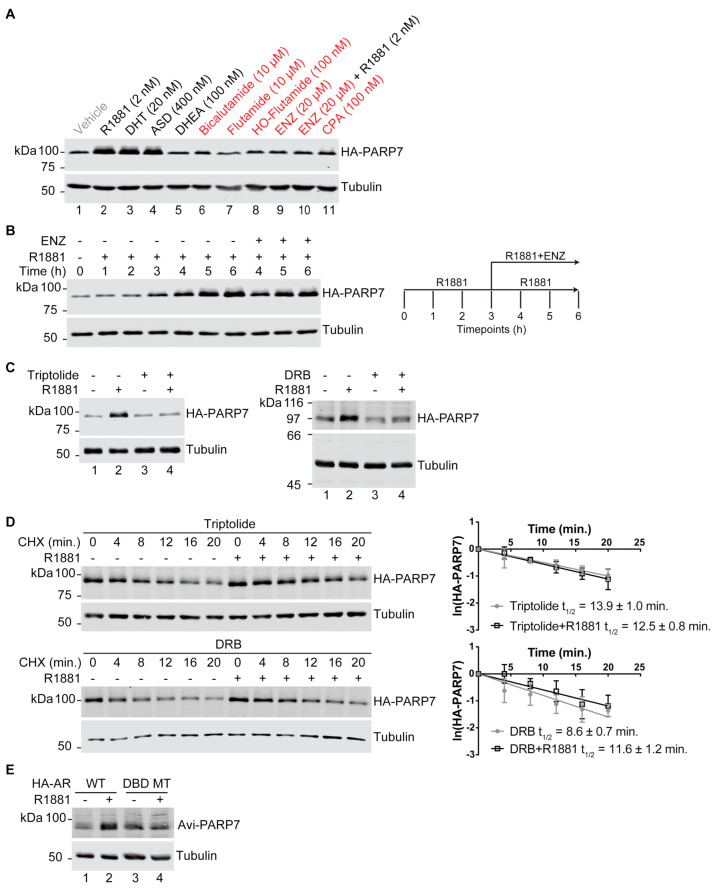
AR-dependent transcription is required for PARP7 stabilization. (**A**) PC3-Flag-AR/HA-PARP7 cells were treated with indicated androgens (black) and anti-androgens (red) for 6 h and analyzed by immunoblotting. DHT: dihydrotestosterone, ASD: androstenedione, DHEA: dehydroepiandrosterone, HO-Flutamide: hydroxyflutamide, ENZ: enzalutamide, CPA: cyproterone acetate. (**B**) PC3-Flag-AR/HA-PARP7 cells were treated with R1881 (2 nM) for 3 h, and then treated for an additional 3 h with R1881 in the presence or absence of ENZ (20 µM) (schematic). Harvested timepoints were analyzed by immunoblotting. (**C**) PC3-Flag-AR/HA-PARP7 cells were pre-treated with transcription inhibitors triptolide (1 μM) or DRB (100 μM) for 1 h, followed by 6 h of androgen (2 nM R1881) treatment and analyzed by immunoblotting. (**D**) PC3-Flag-AR/HA-PARP7 cells were treated as described in (C). Cycloheximide (CHX) time course experiments were carried out under the indicated conditions and analyzed as described in Figure 2A. Plots show mean ± SD from three independent experiments and protein half-lives. (**E**) Avi-tagged PARP7 was co-expressed in PC3M cells stably expressing HA-tagged AR (either wildtype [WT] or V582F mutant which targets the DNA binding domain [DBD MT]). Cells were treated with androgen (2 nM R1881, 6 h) and analyzed by immunoblotting.

Because androgen signaling through AR increases PARP7 protein stability, we reasoned that AR-dependent gene transcription is required for the stabilizing effect on PARP7 protein. For genes positively regulated by AR, transcription is usually dependent on induction of an “agonist conformation”. By contrast, AR activity can be inhibited by compounds such as anti-androgens that induce an “antagonist conformation”. We treated PC3-Flag-AR/HA-PARP7 cells (EF1α promoter) with a panel of agonists and antagonists and performed immunoblotting to query the effects on PARP7 protein stability. With the exception of DHEA (an androgen precursor that has weak AR agonist activity [38]), all of the agonists caused an increase in HA-PARP7 protein level (Figure 6A, compare lane 1 with lanes 2–4). By contrast, none of the anti-androgens increased HA-PARP7 protein level (Figure 6A, compare lane 1 with lanes 6–9 and 11). Enzalutamide (ENZ), which is widely used to treat PCa patients, almost completely blocked the effect of R1881 on HA-PARP7 protein stability (Figure 6A, compare lane 1 and 10). From these data, we conclude that the agonist conformation of AR is required to observe a stabilizing effect on PARP7 protein.

We then conducted a time course of androgen treatment and monitored changes in PARP7 protein level. We found that 3–4 h of androgen treatment is required for the PARP7 protein level to increase (Figure 6B). When ENZ was added to cells after 3 h of R1881 treatment (10000-fold excess), the PARP7 protein level continued to rise (4–6 h, Figure 6B). The length of time required for PARP7 protein stabilization to occur makes it unlikely that PARP7 is stabilized simply through binding agonist-AR because cell entry and androgen binding to AR occurs on a timescale of minutes. Furthermore, if the agonist-induced AR-PARP7 complex was the basis for PARP7 stabilization, then ENZ addition should disrupt the complex and result in PARP7 degradation. The lag period for protein stabilization following androgen treatment and the insensitivity of the stabilization mechanism to ENZ after androgen treatment are better explained by a post-transcriptional mechanism involving AR-dependent transcription of a gene product that promotes PARP7 stabilization.

To address whether the androgen effect on PARP7 protein stability involves AR-dependent transcription, we tested the Pol II transcription inhibitors triptolide and DRB for effects on PARP7 stability. Triptolide and DRB both prevented the R1881-dependent accumulation of HA-PARP7 (Figure 6C, compare lane 2 and 4 within their respective panels). PARP7 protein half-life measurements performed under these conditions showed that the Pol II transcription inhibitors triptolide and DRB blunted androgen-dependent stabilization of PARP7 (Figure 6D).

As an independent test of whether AR-dependent transcription is important for the androgen effect on PARP7 protein stability, we employed PC3M (a highly metastatic variant of PC3) cell lines that express PARP7 with a different epitope tag (Avi), together with HA-tagged forms of WT and mutant AR. The mutant AR selected for this analysis contains a point mutation (V582F) in the DNA binding domain that eliminates dimerization and transcription as measured by an androgen response element promoter luciferase assay [39,40]. When the PC3M-HA-AR/Avi-PARP7 cells were treated with R1881, we observed a stabilizing effect of R1881 cells expressing WT but not mutant AR (Figure 6E; compare lane 1 and 2 with lanes 3 and 4). Thus, multiple approaches establish that agonist-bound, transcriptionally active AR drives PARP7 protein stabilization in a mechanism that is separable from AR regulation of *PARP7* transcription. Our data suggests that that a gene product induced by AR is directly or indirectly involved in PARP7 protein stabilization.

### 3.7. Characterizing In Vivo Role of PARP7 in Mouse Prostate

**Figure 7:**
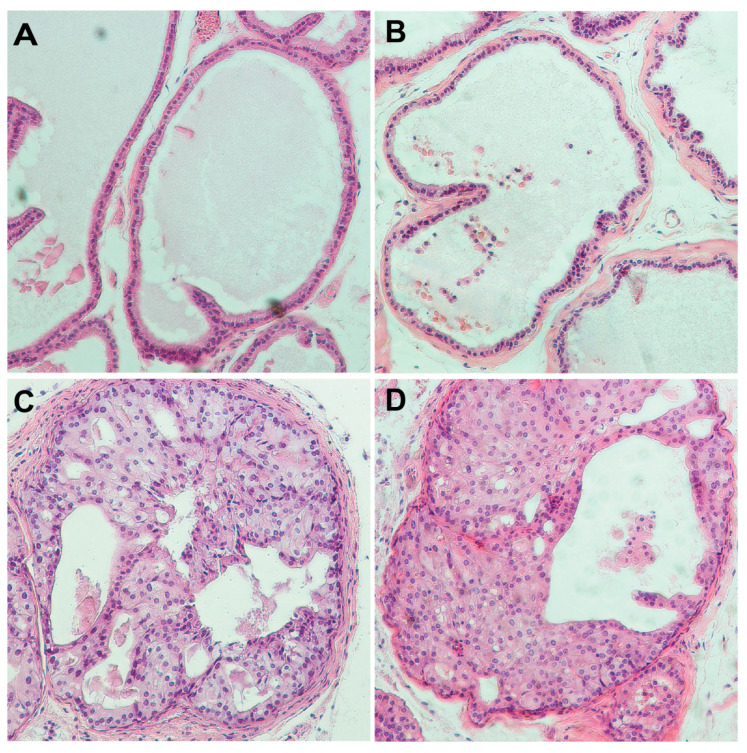
Characterization of PARP7 in a mouse prostate cancer model. Sections of ventral prostates from mice with the four indicated genotypes stained by H&E. Images were captured using a 10× objective. The ages of the mice were as follows: (**A**) WT: 32 weeks, (**B**) *Tiparp^ff^;PbCre4^+^*: 45 weeks, (**C**) *Pten^ff^;PbCre4^+^*: 33 weeks, (**D**) *Pten^ff^;Tiparp^ff^;PbCre4^+^*: 33 weeks.

To examine the function of PARP7 (encoded by the *Tiparp* gene) in prostate, we generated *Tiparp* (*Tiparp^tm1a(EUCOMM)Wtsi^*) mutant mice using cryopreserved sperm obtained from the Canadian Mutant Mouse Repository, Hospital for Sick Children, Toronto. Homozygous *Tiparp* mutant mice have been reported to have reduced viability [41]. In addition, the IMPC reports pre-weaning lethality of C57BL/6N mice homozygous for the *Tiparp^tm1b^* allele, although the penetrance was incomplete (www.mousephenotyping.org). When we intercrossed mice heterozygous for this knock-out first *Tiparp^tm1a^* allele, we obtained 30 mice from six litters that were all either WT or *Tiparp* heterozygous (Chi^2^ *p* < 0.005), consistent with reduced viability in a mixed strain background. We next converted this allele to a conditional allele by breeding with a Flpo-expressing mouse line [31], and examined whether Tiparp levels impact PCa progression in a *Pten^-/-^* background. *Pten* is frequently mutated or lost in human PCa, and has been combined with other mutations to study PCa disease progression [42]. To test if reduced Tiparp levels altered PCa progression in the *Pten* mutant model, we combined a conditional allele of *Tiparp* (referred to as *Tiparp^ff^*) with the conditional *Pten* allele and *PbCre4* to generate prostate epithelium-specific deletion, as in [32]. Mice with homozygous deletion of *Tiparp* in the prostate epithelium appeared normal, as there were no differences in prostate morphology compared to WT mice to an age of 45 weeks (Figure 7A,B). Homozygous deletion of *Pten* from the prostate epithelium results in the onset of high-grade prostate intra-epithelial neoplasia (HGPIN) beginning ~8 weeks of age that progresses slowly to invasive cancer, with HGPIN being the major phenotype in the majority of animals until at least 30 weeks of age [32,33]. After HGPIN, there is an onset of focally invasive cancer, followed by widespread poorly differentiated adenocarcinoma with advancing age. We examined the phenotypes of 14 prostate specific *Pten;Tiparp* double null mice over an age range of 9 to 50 weeks. Below 30 weeks all mice had HGPIN, and among the nine mice examined between 30 and 50 weeks of age, we found similar proportions of focal and more extensive invasive cancer (3 with HGPIN, 4 with focal invasion, and 2 with poorly differentiated adenocarcinoma) to those seen in *Pten* null mice. In addition to the lack of difference in the proportions of invasive cancer and HGPIN, we did not observe any additional phenotypes in the *Tiparp* mutants, and HGPIN in *Pten;Tiparp* mutants was not different from that in *Pten* nulls (Figure 7C,D). Thus, it appears that in this strain background, loss of *Tiparp* alone does not affect prostate morphology, and loss of Tiparp does not detectably affect the *Pten* null prostate tumor phenotype.

## 4. Discussion

PARP7 has been studied in the context of breast [22,23], ovarian [24,25], and colon cancer [23], but the information on PARP7 in PCa cells is very limited. We found that in PCa cells, PARP7 undergoes very rapid turnover in a proteasome-dependent manner (Figure 2 and Figure 3). We determined that PARP7 has a half-life of ~4.5 min, making it one of the most rapidly degraded proteins in the cell. The half-life of PARP7 is comparable to several other short-lived proteins such as Cyclin D1 (t_1/2_ = 24–30 min) [43,44], TRIM52 (t_1/2_ = 3–3.5 min) [45], HIF1-alpha (t_1/2_ = 4–8 min) [46,47], and ornithine decarboxylase (t_1/2_ = 5–30 min) [48]. The rapid turnover of PARP7 likely extends to other cell types given that MG132 treatment of HuH-7 hepatocarcinoma resulted in a significant accumulation of PARP7 [16]. This suggests that PARP7 instability might not be dependent on cell context; we propose that the rapid degradation of PARP7 is likely mediated by intrinsic features of the protein structure working in concert with cellular machinery.

A second major finding from our study is that PARP7 is stabilized by AR signaling which increases its half-life from approximately 4.5 to 25.2 min (Figure 2). Through multiple approaches using various AR ligands, Poll II inhibitors, and an AR DNA binding domain mutant, we found that AR-dependent transcription is required for the stabilization of PARP7 and subsequent accumulation of the protein (Figure 6). While it was known previously that AR signaling induces *PARP7* mRNA, our analysis clearly shows that protein stabilization is a second layer of regulation by AR signaling for PARP7. PARP7 protein stabilization could reflect androgen regulation of the ubiquitylation machinery, including E3 ubiquitin ligases and deubiquitinases, or an effect that reduces PARP7 utilization as a substrate for degradation. Given that a ~4-fold increase in PARP7 protein level is observed by androgen treatment (Figure 4B), AR-driven stabilization is likely to have a significant amplifying effect on PARP7 protein expression, for a given amount of *PARP7* mRNA induction. It was somewhat surprising that deletion of *PARP7* did not generate a discernable phenotype in prostate where androgen signaling is critical for normal glandular function and cancer development.

Protein instability described for PARP7 does not appear to be a general feature of PARPs at least based on a limited sampling of several family members. PARP1, PARP2, PARP9, and PARP14 proteins were relatively stable and unaffected by cycloheximide and MG132 treatment within the time frame of our experiments (Figure 4). Sequence analysis using IUPred2A [49], an online bioinformatics tool for prediction of disordered protein regions, showed that the N-terminus of PARP7 (approximately the first 170 amino acids) is intrinsically disordered, while interestingly PARP1, PARP9, and PARP14 lacked such regions. Intrinsically disordered regions within proteins can have a destabilizing effect and promote rapid degradation [50,51]. Thus, the instability differences between PARP7 versus PARP1, PARP9, or PARP14 might therefore be explained at least in part by the presence or absence of intrinsically disordered regions. While PARP2 has a natively disordered N-terminal region [52], it appeared relatively stable in our assays. We note that the zinc finger domain and WWE domain which are not found in PARP2, as well as catalytic function of PARP7 are required for its rapid turnover properties. This emphasizes that multiple structural aspects of PARP7 contribute to its instability feature.

We found that the catalytic function of PARP7 is linked to its rapid turnover as introducing loss-of-function mutations (H532A or Y564A) into the catalytic domain increased the half-life from ~4.5 min in wild type to ~34.0–40.7 min in the mutants (Figure 3). PARP7 is known to undergo auto-modification [7,16], and thus, ADP-ribosylation could serve as a targeting signal for degradation, which is consistent with the protein stabilizing effect of the catalytic domain mutations. A precedent for linking ADP-ribosylation with protein degradation exists: in the Wnt signaling pathway, the Axin protein is poly-ADP-ribosylated by tankyrases and targeted for degradation by RNF146 [53]. The fact that the very rapid turnover of PARP7 depends on the catalytic function raises the interesting idea that ADP-ribosylation-dependent, constitutive degradation of PARP7 is a self-limiting mechanism for PARP7 in cells. Consistent with this view, a recent study found that ubiquitylation of PARP7 depends on the catalytic function of the protein, although the specific degradation mechanism for PARP7 was not defined [23].

We also found that the zinc finger domain of PARP7 makes a significant contribution to its unstable nature. Single mutations (C243A and C251A) within the zinc finger resulted in a significant increase in protein half-life (Figure 3). Among all the mutants that we tested, the increase in PARP7 stability was the largest for the zinc finger mutations, suggesting that this domain makes relatively large contributions to the turnover of PARP7. Based on our data, it is plausible that the PARP7 zinc finger domain acts as a recognition motif for the cellular machinery responsible for PARP7 degradation. A proteome-wide survey showed that K259 within the PARP7 zinc finger undergoes ubiquitylation [36]. The deletion of the WWE domain also had an increase in PARP7 protein half-life, and along the same line of reasoning as the zinc finger domain, we speculate that the WWE domain may also be a recognition motif for protein degradation.

Understanding both the transcriptional and post-transcriptional mechanisms that control PARP7 levels may have clinical relevance given the recent data suggesting PARP7 may impact patient survival for breast and ovarian cancer [23,25]. These analyses on clinical samples were carried out based on *PARP7* mRNA levels, and in light of our new findings, it becomes clear that simply examining mRNA levels may not provide a complete picture of the PARP7 expression status, as the amount of PARP7 could be significantly impacted by how PARP7 protein turnover is regulated in those settings. To address this question, it will be important that future studies fully characterize the PARP7 degradation machinery and how androgen signaling modulates this mechanism. The regulatory factors involved in PARP7 degradation may be clinically useful as biomarkers and provide a fuller picture of PARP7 status in patients. In a genetically engineered mouse model for PCa, we did not detect an effect of deleting PARP7 in Pten-dependent tumorigenesis. Characterizing the settings where PARP7 contributes to tumorigenesis, and how this is integrated with its rapid turnover, is an important challenge. In PCa cells, AR signaling drives stabilization of PARP7 and subsequent accumulation within the nucleus, and in order to understand the nuclear roles of PARP7, it is important to characterize the substrates of PARP7 and the functional outcome of PARP7-mediated mono-ADP-ribosylation. Ultimately, understanding PARP7 biogenesis and the pathways controlled by PARP7 could reveal the context where manipulation of PARP7 can improve outcomes in certain cancers.

## Figures and Tables

**Table 1:** Summary of PARP7 protein half-lives.

PARP7	t_1/2_ (Vehicle)	t_1/2_ (R1881)
Endogenous PARP7	N/A ^1^	12 h R1881: 30.7 ± 2.8 24 h R1881: 27.1 ± 1.8
HA-PARP7 WT	4.5 ± 0.1	25.2 ± 1.5
C243A	99.1 ± 7.4	74.9 ± 8.8
C251A	99.0 ± 10.5	90.3 ± 9.2
H532A	40.7 ± 3.5	42.0 ± 2.5
Y564A	34.0 ± 1.6	33.0 ± 1.4
∆WWE	14.2 ± 0.8	14.0 ± 0.7

^1^ In the absence of androgen (R1881) treatment, endogenous PARP7 expression is undetectable by immunoblotting, precluding determination of a protein half-life. Unless indicated otherwise, R1881 treatment was conducted for 6 h. Protein half-lives (minutes) are shown as mean ± SD from three biological replicates. For analysis of mutant PARP7 protein half-lives, see Figure S1.

## Data Availability

Source data available upon request.

## Supplementary Materials

The following are available online at https://www.mdpi.com/2073-4409/10/2/363/s1, Figure S1: Analysis of protein half-lives for PARP7 mutants in the presence of androgen, Figure S2: Targeted disruption of the *Tiparp* gene in mouse prostate using Cre recombinase.
