# Supplementary material for: Post-Transcriptional Regulation of PARP7 Protein Stability Is Controlled by Androgen Signaling
Source: Cells. 2021 Feb 9;10(2):363. doi: 10.3390/cells10020363 (PMC7916378; doi:10.3390/cells10020363)
Supplement: Supplementary file 1 [file cells-10-00363-s001.pdf]

## Supplementary Materials

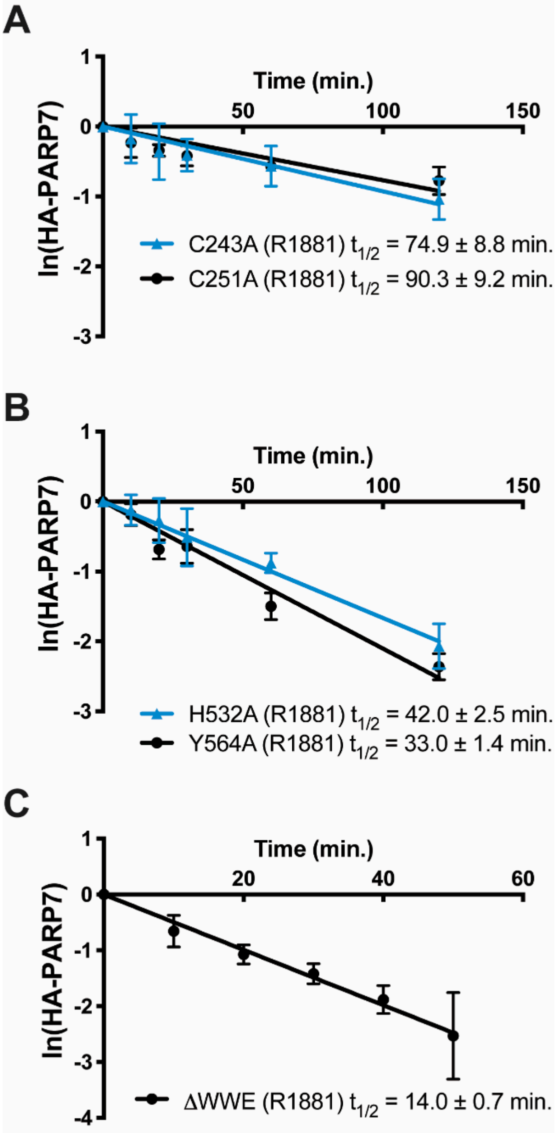


**Supplementary Figure S1.** Analysis of protein half-lives for PARP7 mutants in the presence of androgen. (**A**) PARP7 zinc finger mutants (C243A or C251A), (**B**) catalytic mutants (H532A or Y564A), or (**C**) WWE deletion mutant (ΔWWE) was induced in PC3-Flag-AR cells by doxycycline treatment (2 μg/mL, 24 h). Following doxycycline treatment, cells were treated with androgen (2 nM R1881, 6 h). Cycloheximide (CHX) time course experiments were conducted and analyzed as described in Fig 2A. Plots show mean ± SD (n = 3) and protein half-lives.

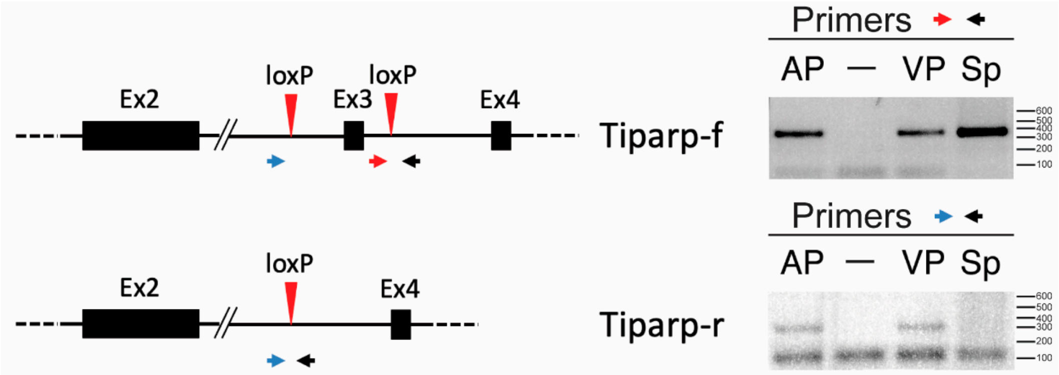


**Supplementary Figure S2.** Targeted disruption of the *Tiparp* gene in mouse prostate using Cre recombinase. Schematic of the conditional Tiparp allele before (upper) and after (lower) recombination. LoxP sites (red triangles) flank exon 3, which is removed by Cre-mediated excision. Positions of PCR primers used to detect the loxP (-f) allele and the recombined (-r) allele are shown (red, blue and black arrows). The red+black primer pair detects the f allele and the blue+black pair detect the r allele. PCR analysis of anterior and ventral prostate (AP and VP) and spleen (Sp) for the f and r alleles is shown (right). Prostate-specific Cre expression is driven by the probasin promoter [1].

**Reference**

1. Maddison, L.A.; Nahm, H.; DeMayo, F.; Greenberg, N.M. Prostate Specific Expression of Cre Recombinase in Transgenic Mice. *genesis* **2000**, *26*, 154–156, doi:10.1002/(SICI)1526-968X(200002)26:2<154::AID-GENE18>3.0.CO;2-
